# What do the voice-related parameters tell us? The multiparametric index scores, cepstral-based methods, patient-reported outcomes, and durational measurements

**DOI:** 10.1007/s00405-024-09192-w

**Published:** 2025-01-19

**Authors:** Hakan Gölaç, Adnan Gülaçtı, Güzide Atalık, Gözde Bayramoğlu Çabuk, Metin Yılmaz

**Affiliations:** 1 Department of Speech and Language Therapy, Faculty of Health Sciences, Gazi University, Ankara, Türkiye; 2 Department of Otolaryngology, Faculty of Medicine, Gazi University, Ankara, Türkiye

**Keywords:** Dysphonia severity, Multiparametric indexes, Smoothed cepstral peak prominence, Patient-reported outcomes, Durational measurements

## Abstract

**Purpose:**

This study aimed to identify how the acoustic parameters, patient-reported outcomes (PROs), and durational measurements differ based on perceptually rated dysphonia severity and to investigate their relationship with dysphonia severity.

**Methods:**

One hundred seventy-nine subjects (males-78, females-101; mean ± SD age of 47.79 ± 14.05 years) with various etiology of dysphonia were included in this prospective cohort study. The *G* parameter of GRBAS was used to rate dysphonia severity. The Acoustic Voice Quality Index (AVQI v. 03.01), Acoustic Breathiness Index (ABI), and cepstral peak prominence-smoothed (CPPS) values for sustained vowel (CPPS*sv*) and connected speech (CPPS*cs*) samples were obtained using the *Praat* software. The Voice Handicap Index-10 (VHI-10) and Voice-Related Quality of Life (V-RQOL) were used for PROs, and the maximum phonation time (MPT) and s/z ratio were measured as durational parameters.

**Results:**

The acoustic parameters, including AVQI and ABI scores, and CPPS*sv* and CPPS*cs* values significantly differed based on dysphonia severity, particularly in those with moderate (G_2_) or severe dysphonia (G_3_) compared to those with normal (G_0_) and/or slightly deviated (G_1_) voice. Among the PROs, VHI-10 scores significantly differed only between the groups G_1_ and G_2_ (*p* < 0.005) and the groups G_1_ and G_3_ (*p* < 0.005), whilst there were not any significant differences between the V-RQOL scores of all the compared groups. Significant differences were observed within the groups for MPT, but the s/z ratio significantly differed only between the groups G_1_ and G_3_. Correlation analysis revealed significant relationships between the *G* parameter of GRBAS and all measured variables (*p* < 0.001).

**Conclusions:**

Particular attention should be paid to the AVQI and ABI scores, CPP*sv* and CPP*cs* values, VHI-10 scores, and MPT values since they provide valuable information for overall dysphonia severity.

## Introduction

A comprehensive voice assessment protocol should carefully consider all aspects of voice, including videolaryngostroboscopic examination, auditory-perceptual assessments, acoustic voice analyses, patient-reported outcomes (PROs), and respiratory/aerodynamic measurements. In the current procedures from different parts of the world, incorporating both subjective and objective types of voice assessments are recommended to identify the underlying pathophysiology of dysphonia and to build up appropriate intervention and management therapy programs [1–4].

Videolaryngostroboscopy is considered the gold standard for visualizing laryngeal function and is used to evaluate various variables such as glottal closure, amplitude of vibration, mucosal wave, vertical level of vocal folds, presence of non-vibrating portions of the vocal folds, supraglottic activity, free edge contour, phase closure and symmetry of the movements, and regularity [5–8].

Acoustic voice analyses are among the commonly used objective voice quality measurements since they detect subtle voice changes that remain inaudible to the human ear [9]. Recent studies have shown that instead of the conventional perturbation measurements such as jitter, shimmer, and noise to harmonic ratio (NHR), cepstral parameters – i.e., cepstral peak prominence (CPP) and cepstral peak prominence-smoothed (CPPS) – can be used for assessing the severity of dysphonia with acceptable feasibility, reliability, and validity [10–13]. Further, multiparametric models that reflect the multidimensional nature of voice, such as the Dysphonia Severity Index (DSI) [14], the Cepstral Spectral Index of Dysphonia (CSID) [15, 16], the Acoustic Voice Quality Index (AVQI) [17], the Acoustic Breathiness Index (ABI) [18], and the Voice Wellness Index (VWI) [19] have increasingly supported in many studies [20–24], because they combine various voice-related parameters to measure the dysphonia severity.

Using valid, reliable, and responsive PROs is one of the main parts of voice assessment, because of providing a method of systematically capturing patient’s experiences and perspectives on their voice. Since they reflect the patient’s experiences and perspectives on their voice, they have the potential to facilitate patient involvement in treatment decision-making and provide guidance for health-care decisions [25, 26]. Recently, many voice-related PROs – i.e., the Voice Handicap Index-10 (VHI-10) and the Voice-Related Quality of Life (V-RQOL) – are available worldwide [26–28], which can be used for various dysphonia etiologies in specific patient groups, and their usage has gained popularity in both the clinical and research milieus [27].

Durational parameters of voicing – i.e., the maximum phonation time (MPT) and s/z ratio – provide valuable information about the interaction between respiratory and phonatory mechanisms of the speech system [29–31]. Therefore, they have been widely used as a simple clinical test to distinguish the disordered voice from a normal voice in voice clinics, given the nature of being non-invasive, easy to perform, fast feasibility, and low cost [32–35].

Since voice quality is a perceptual phenomenon in voice, auditory-perceptual assessment plays a crucial role in the clinical evaluation of patients with dysphonia [36]. Even if auditory-perceptual assessment has been shown to be unreliable across the continuum of clinical experience [37], it is still considered as the “gold standard” procedure for defining a disordered voice [37–39]. Among some specifically designed international auditory-perceptual evaluation schemes or scales [36], GRBAS (grade, roughness, breathiness, asthenia, strain) scale [40], and the Consensus Auditory-Perceptual Evaluation of Voice (CAPE-V) [41] are the most frequently reported and most accepted rating methods in the auditory-perceptual evaluation of voice [42]. Currently, these methods are commonly used in clinical and speech research settings to differentiate normal voice from disordered voice [37–39, 43].

As mentioned above, the voice assessment is a multidimensional approach requiring both objective and subjective outcomes of the patient’s voice [1–4]. Monodimensional approaches that combine some but not all of the following dimensions: videolaryngostroboscopic examination, auditory-perceptual assessment, acoustic voice analyses, PROs, and durational measurements are not recommended, because these dimensions may be independent from one to another [3, 44]. However, the number of studies that evaluate all voice-related dimensions together on the same cohort of patients with dysphonia is limited [3, 34, 45]. Therefore, comprehensive voice evaluation studies considering all voice-related aspects are needed to reveal the general voice characteristics of patients with dysphonia.

In this comprehensive voice evaluation study, we primarily aimed to identify how the acoustic parameters, PROs, and durational measurements differ based on the dysphonia severity, which was determined using auditory-perceptual assessment in a cohort of patients with various etiology of dysphonia. Further, the relationships between dysphonia severity and multiparametric index scores (AVQI and ABI), cepstral-based methods (CPPS), PROs (VHI-10 and V-RQOL), and durational measurements (MPT and s/z ratio) were investigated to figure out how these voice-related dimensions correlate with auditory-perceptual assessment.

## Materials and methods

### Study design and ethical consideration

This study was designed as a prospective cohort study that included voice samples of patients with voice disorders assessed at our routine dysphonia clinical practice. Ethical approval was obtained from the research ethics committee at Gazi University (registration number: 254).

### Participants

In total, 179 subjects (males-78, females-101) with a mean age ± standard deviation (SD) of 47.79 ± 14.05 years were examined at Prof. Dr. Necmettin Akyıldız Hearing, Speech, and Voice Center. All subjects were consecutively examined by an experienced laryngologist and a speech and language pathologist (SLP).

As a routine part of our clinical voice assessment procedure, the following protocol was applied for each patient with dysphonia:

- Videolaryngostroboscopic examination.
- Auditory-perceptual assessment.
- Acoustic voice analyses.
- Measurements of PROs.
- Durational measurements, including MPT and s/z ratio.

Patients were diagnosed after the above-mentioned comprehensive voice assessment protocol. After the diagnostic procedure, all subjects were referred for medical intervention and/or behavioral voice therapy. The general demographics and dysphonia etiologies of the included subjects are outlined in Table 1.

**Table 1:** General characteristics of the participants

Parameters	Study population, *N* = 179 (100%)
Age in years, mean ± SD	47.79 ± 14.05
Gender, N (%)	
Female Male	101 (56.4) 78 (43.6)
Diagnosis, N (%)	
Functional dysphonia Paralysis/paresis Nodules Sulcus vocalis Cysts Post-cordectomy Refluxlaryngitis Reinke’s edema Muscle tension dysphonia Post-radiotherapy Spasmodic dysphonia Mutational falsetto Glottal insufficiency Polyp Leukoplakia Presbylarynx Granuloma Laryngeal trauma Laryngitis	26 (14.5) 19 (10.6) 17 (9.5) 15 (8.4) 14 (7.8) 14 (7.8) 11 (6.1) 11 (6.1) 9 (5.0) 8 (4.5) 7 (3.9) 7 (3.9) 7 (3.9) 5 (2.8) 4 (2.3) 2 (1.1) 1 (0.6) 1 (0.6) 1 (0.6)

*Abbreviations*: N, number; SD, standard deviation; %, percentage

Voice samples of professional voice users, those with any other problems that limited communication, subjects who could not tolerate the videolaryngostroboscopic examination, and those who were uncooperative for voice recordings were excluded from the dataset of the present study.

### Voice records

All voice samples were recorded in a sound-treated room with an ambient noise level of 30 ± 5 dBC using the computerized speech laboratory (CSL) software (Model 3700, Version 3.4.1, 2000–2001 Kay PENTAX, Montvale, NJ). A unidirectional Rode NT1 cardioid condenser microphone (Rode microphones, Torrance, CA) with a frequency response of 20 Hz to 20 kHz (± 2 dB) was positioned at a fixed mouth-to-microphone distance of 15 cm. During the voice recording process, all individuals were first asked to produce a sustained [a: ] at a comfortable pitch and loudness for at least 5 s and then to read a standard reading passage (Ömer Seyfettin’s novel titled “Diyet”). Before the recordings, the second author (A.G.) illustrated all the procedures to each participant. The recorded voice signals were saved in an uncompressed.*wav* format with a sampling rate of 44.100 Hz and a 16-bit resolution for further acoustic analyses.

### Acoustic analyses

Acoustic analyses of the recorded voice samples were obtained using the Praat software (Version 6.3.20, Paul Boersma and David Weenink, Department of Phonetic Sciences, University of Amsterdam, Amsterdam, Netherlands).

For both AVQI (v. 3.01) and ABI measurements, the mid-vowel 3 s of the previously recorded vowel /a/ was used for the sustained vowel (*sv*). The customized length of continuous speech procedure described by Barsties and Maryn [46] was used for the continuous speech (*cs*) segment. Based on this procedure, the first paragraph of the reading passage was used after removing all voiceless segments with a modified Praat-script developed by Kılıç [47, 48]. This modified Praat-script also provides equalization of the *sv* and *cs* segments to 3.00 s to eliminate the need to count syllables and the impact caused by reading speed. The 3 s of the *sv* and voiced *cs* segments were chained to realize a higher level of ecological validity both for AVQI and ABI measurements [17]. Also, the CPPS values were extracted for both *sv* and *cs* segments as CPPS*sv* and CPPS*cs* using the same script [47].

### Auditory-perceptual assessment

Auditory-perceptual assessments were obtained on the concatenation of the *sv* and the *cs* without removing voiceless segments. An external primary rater (an SLP with at least 10 years of experience in evaluating and measuring voice disorders) blindly assessed all voice samples. After two weeks of interval, 20% of the data were randomly selected, and repeated judgments were conducted by the primary rater and an external second rater (an SLP with at least 5 years of experience in evaluating and measuring voice disorders) to capture intra- and inter-rater reliability. Each rater judged the overall dysphonia grade, the *G* (Grade) parameter of the GRBAS scale [40], using a four-point ordinal scale (i.e., 0 = normal, 1 = slightly hoarse or breathy, 2 = moderately hoarse or breathy, and 3 = severely hoarse or breathy). All voice samples were presented to the raters in a sound-treated room using headphones, and they were allowed to re-listen to the voice samples at any time they wanted before making a final decision.

### Patient-reported outcomes

Both Turkish versions of VHI-10 [49] and V-RQOL [50] questionnaires were used as PROs, developed initially by Rosen et al. [51] and Hogikyan and Sethuraman [52], respectively. The VHI-10 is known as a valid and reliable tool measuring the handicap of voice on patients with a rating scale from “0” (never) to “4” (always) for each of the 10 items. The higher score means greater voice-related handicap, and vice versa. The other questionnaire that we used, V-RQOL, also has 10 items, rating from “1” (none, not a problem) to “5” (as bad as it can be). It measures the quality of life rather than handicap and has a standardized scoring algorithm ranging from 0 to 100, with a higher score indicating a better voice-related quality of life in patients with dysphonia.

### Durational measurements

As durational measurements, MPT and s/z ratio were collected by an SLP. First, patients were instructed to take a deep breath and sustain the /a/, /s/, and /z/ phonemes in a comfortable pitch and loudness as long as possible. Then, the longest duration of three trials for each phoneme was noted. The longest duration of sustained /a/ phonation was used for the MPT, whereas the longest duration of sustained /s/ phonation divided by the longest duration of sustained /z/ phonation was used for the s/z ratio.

### Statistical analyses

All data analyses were completed using the Statistical Package for Social Sciences (SPSS Inc. Chicago, USA, version 25.0). Comparisons between the groups that were determined based on dysphonia severity (G_0_, G_1_, G_2_, and G_3_) were performed using the Kruskal-Wallis test for all measured variables (AVQI and ABI scores, CPPS*sv* and CPPS*cs*, VHI-10 and V-RQOL scores, and MPT and s/z ratio). In the case where a significant difference was found between the four groups, then the pairwise comparisons were performed with the Bonferroni adjusted Mann-Whitney U test. The relationships between the above-mentioned variables and the *G* parameter of the GRBAS scale were examined with the Spearman’s correlation test. Intra- and inter-rater reliabilities for the *G* parameter of the GRBAS scale were calculated using the Cohen’s Kappa value. The kappa results were interpreted as follows: < 0; no agreement, 0.01–0.20; slight agreement, 0.21–0.40; fair agreement, 0.41–0.60; moderate agreement, 0.61–0.80; substantial agreement, and 0.81–0.99, almost perfect agreement [53]. All the performed tests were two-tailed, and the statistical significance level was set at *p* < 0.05 in this study.

## Results

Across the entire cohort, the mean ± SD was 4.27 ± 1.82 for AVQI score, 5.56 ± 1.47 for ABI score, 12.53 ± 3.01 for CPPS*sv*, 9.91 ± 1.87 for CPPS*cs*, 18.80 ± 10.96 for VHI-10 score, 53.72 ± 28.25 for V-RQOL score, 11.27 ± 5.89 for MPT, and 1.19 ± 0.64 for s/z ratio. Number of the subjects in G_0_, G_1_, G_2_, and G_3_ were *N* = 19 (10.6%), *N* = 73 (40.8%), *N* = 51 (28.5%), and *N* = 36 (20.1%), respectively (Table 2).

**Table 2:** Results of the measured variables across all participants and the number (%) of the subjects based on dysphonia grade

Variables	Mean ± SD (Range)
AVQI score	4.27 ± 1.82 (1.06–9.79)
ABI score	5.56 ± 1.47 (2.44–9.87)
CPPS*sv* (dB)	12.53 ± 3.01 (3.83–18.46)
CPPS*cs* (dB)	9.91 ± 1.87 (3.98–13.94)
VHI-10 score	18.80 ± 10.96 (0–40)
V-RQOL score	53.72 ± 28.25 (0–100)
MPT (s)	11.27 ± 5.89 (2.11–32.43)
s/z ratio	1.19 ± 0.64 (0.44–5.40)
Overall dysphonia grade, N (%)	
0 1 2 3	19 (10.6) 73 (40.8) 51 (28.5) 36 (20.1)

*Abbreviations*: SD, standard deviation; AVQI, acoustic voice quality index; ABI, acoustic breathiness index; CPPS*sv*, cepstral peak prominence-smooted (sustained vowel); dB, decibel; CPPS*cs*, cepstral peak prominence-smooted (connected speech); VHI-10, voice handicap index-10; V-RQOL, voice-related quality of life; MPT, maximum phonation time; s, second; N, number; %, percentage

Reliability analyses showed almost perfect agreement for both the intra- and inter-rater agreements with Kappa values of 0.956 (*p* < 0.001) and 0.863 (*p* < 0.001), respectively. Therefore, the first rater’s scores on the *G* parameter were considered for auditory-perceptual evaluation.

All measured variables, namely the AVQI and ABI scores, CPPS*sv* and CPPS*cs*, VHI-10 and V-RQOL scores, and MPT and s/z ratio significantly differed after the comparisons between the four groups (G_0_, G_1_, G_2_, and G_3_). More detailed data, including the mean, standard deviation (SD), minimum, and maximum values of the related variables are outlined in Table 3.

**Table 3:** Mean, standard deviation (SD), minimum, and maximum values of the measured variables across the overall dysphonia grade

Variables	G_0_ (*N* = 19) Mean ± SD (Range)	G_1_ (*N* = 73) Mean ± SD (Range)	G_2_ (*N* = 51) Mean ± SD (Range)	G_3_ (*N* = 36) Mean ± SD (Range)	*p*-value
AVQI score	2.96 ± 1.01 (1.85–6.22)	3.38 ± 1.01 (1.43–6.25)	4.54 ± 1.46 (1.06–7.55)	6.61 ± 1.83 (1.74–9.79)	< 0.001
ABI score	4.38 ± 1.19 (2.44–7.33)	5.09 ± 0.94 (2.81–7.45)	5.68 ± 1.44 (2.85–8.60)	7.09 ± 1.41 (3.58–9.87)	< 0.001
CPPS*sv* (dB)	14.65 ± 2.08 (8.82–17.45)	13.51 ± 2.09 (6.31–18.46)	12.18 ± 2.85 (6.58–17.72)	9.67 ± 3.29 (3.83–17.01)	< 0.001
CPPS*cs* (dB)	11.06 ± 1.15 (8.52–12.92)	10.92 ± 1.03 (7.89–13.94)	9.60 ± 1.25 (7.19–12.18)	7.50 ± 2.05 (3.98–13.29)	< 0.001
VHI-10 score	13.50 ± 9.96 (0–29)	15.55 ± 9.71 (0–31)	22.22 ± 11.24 (0–40)	22.97 ± 10.77 (5–40)	0.001
V-RQOL score	64.82 ± 27.72 (25–100)	60.93 ± 25.96 (10–100)	46.59 ± 27.71 (0–95)	44.54 ± 29.03 (0–100)	0.012
MPT (s)	14.33 ± 6.26 (5.62–25.66)	13.47 ± 5.55 (5.30–32.43)	9.74 ± 5.15 (4.98–23.86)	7.31 ± 4.48 (2.11–21.10)	< 0.001
s/z ratio	0.99 ± 0.32 (0.44–1.56)	1.08 ± 0.72 (0.48–5.40)	1.26 ± 0.56 (0.57–3.18)	1.82 ± 1.81 (0.55–2.56)	0.003

*Abbreviations*: G, overall dysphonia grade; N, number; SD, standard deviation; AVQI, acoustic voice quality index; ABI, acoustic breathiness index; CPPS*sv*, cepstral peak prominence-smooted (sustained vowel); dB, decibel; CPPS*cs*, cepstral peak prominence-smooted (connected speech); VHI-10, voice handicap index-10; V-RQOL, voice-related quality of life; MPT, maximum phonation time; s, second

**Table 4:** Spearman’s correlation coefficient between the overall dysphonia grade (G) and all measured parameters

Parameters	G (Overall dysphonia grade)	
	*r*	*p*-value
AVQI score	0.642	< 0.001
ABI score	0.518	< 0.001
CPPS*sv* (dB)	-0.497	< 0.001
CPPS*cs* (dB)	-0.646	< 0.001
VHI-10 score	0.319	< 0.001
V-RQOL score	-0.270	< 0.001
MPT (s)	-0.496	< 0.001
s/z ratio	0.308	< 0.001

*Abbreviations*: G, overall dysphonia grade; r, correlation coefficient; AVQI, acoustic voice quality index; ABI, acoustic breathiness index; CPPS*sv*, cepstral peak prominence-smooted (sustained vowel); dB, decibel; CPPS*cs*, cepstral peak prominence-smooted (connected speech); VHI-10, voice handicap index-10; V-RQOL, voice-related quality of life; MPT, maximum phonation time; s, second

**Fig. 1:**
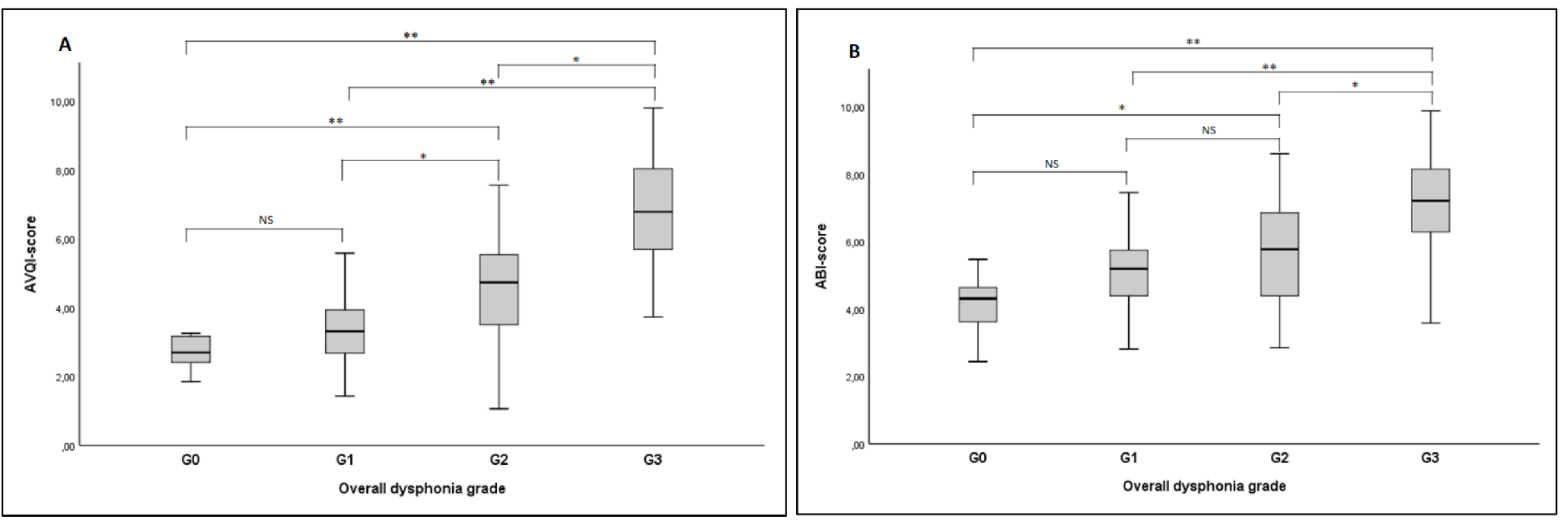
**(A)** AVQI and **(B)** ABI scores based on dysphonia severity *Abbreviations*: AVQI, acoustic voice quality index; ABI, acoustic breathiness index; G, overall dysphonia grade *, *p* < 0.005. **, *p* < 0.001. NS, not significant.

The pairwise comparisons between the groups revealed significantly higher AVQI scores for the G_3_ group compared to the other groups (G_0_; *p* < 0.001, G_1_; *p* < 0.001, and G_2_; *p* < 0.005). Significantly higher AVQI scores were observed in the G_2_ group compared to the groups G_0_ (*p* < 0.001) and G_1_ (*p* < 0.005), but the same was not evident for the comparison between the G_0_ and G_1_ groups. For ABI scores, significantly higher values were detected in the G_3_ group compared to the other groups (G_0_; *p* < 0.001, G_1_; *p* < 0.001, and G_2_; *p* < 0.005). The ABI score in the G_2_ group was significantly higher than the group G_0_ (*p* < 0.005), but no statistically significant difference was found between the ABI scores in groups G_2_ and G_1_. On the other hand, comparisons between the G_0_ and G_1_ groups did not reveal any significant differences (Fig. 1).

**Fig. 2:**
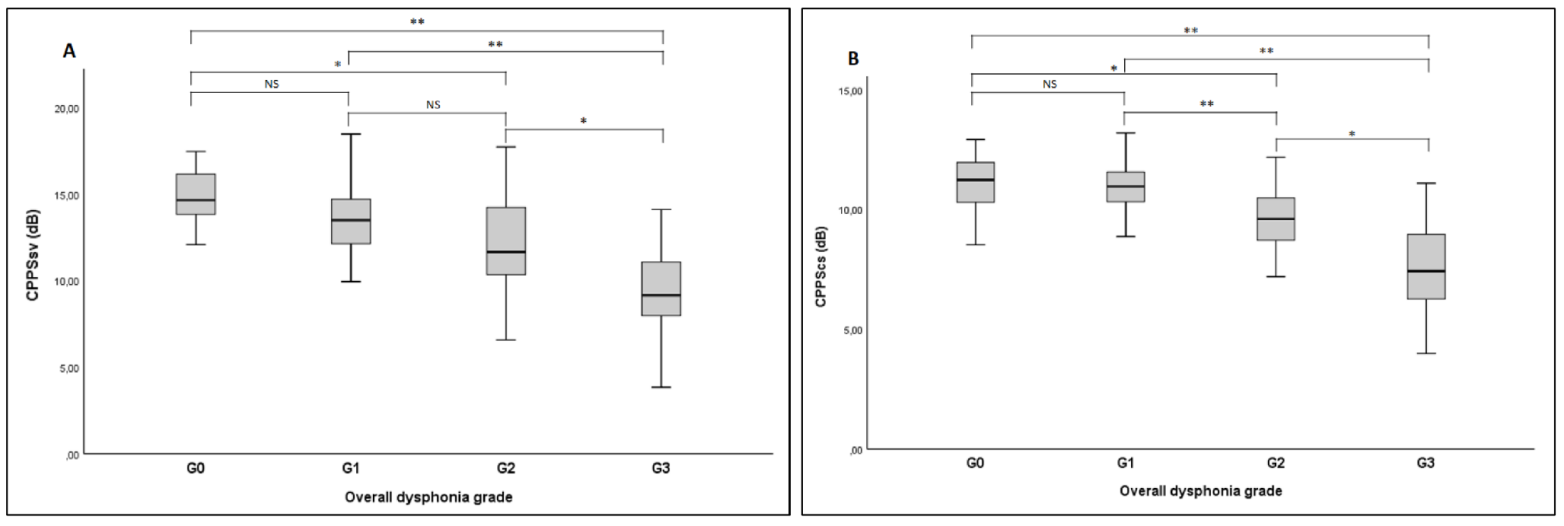
**(A)** CPPS*sv* and **(B)** CPPS*cs* values based on dysphonia severity *Abbreviations*: CPPS*sv*, cepstral peak prominence-smooted (sustained vowel); dB, decibel; CPPS*cs*, cepstral peak prominence-smooted (connected speech); G, overall dysphonia grade *, *p* < 0.005. **, *p* < 0.001. NS, not significant.

In group G_0_, CPPS*sv* values were significantly higher than in groups G_2_ (*p* < 0.005) and G_3_ (*p* < 0.001), whereas no differences were observed in comparisons with group G_1_. The CPPS*sv* values were significantly higher in group G_1_ compared to group G_3_ (*p* < 0.001), but no significant differences were observed between group G_1_ compared to group G_2_. Lastly, higher CPPS*sv* values were found in the group G_2_ compared to the group G_3_ (*p* < 0.005). The comparisons for CPPS*cs* between the groups showed significantly higher values in the G_0_ group than the groups G_2_ (*p* < 0.005) and G_3_ (*p* < 0.001), but not than the group G_1_. The CPPS*cs* values in group G_1_ were found to be higher than both the G_2_ (*p* < 0.001) and G_3_ groups (*p* < 0.001). Lastly, group G_2_ had significantly higher CPPS*cs* values than group G_3_ (*p* < 0.005), (Fig. 2).

**Fig. 3:**
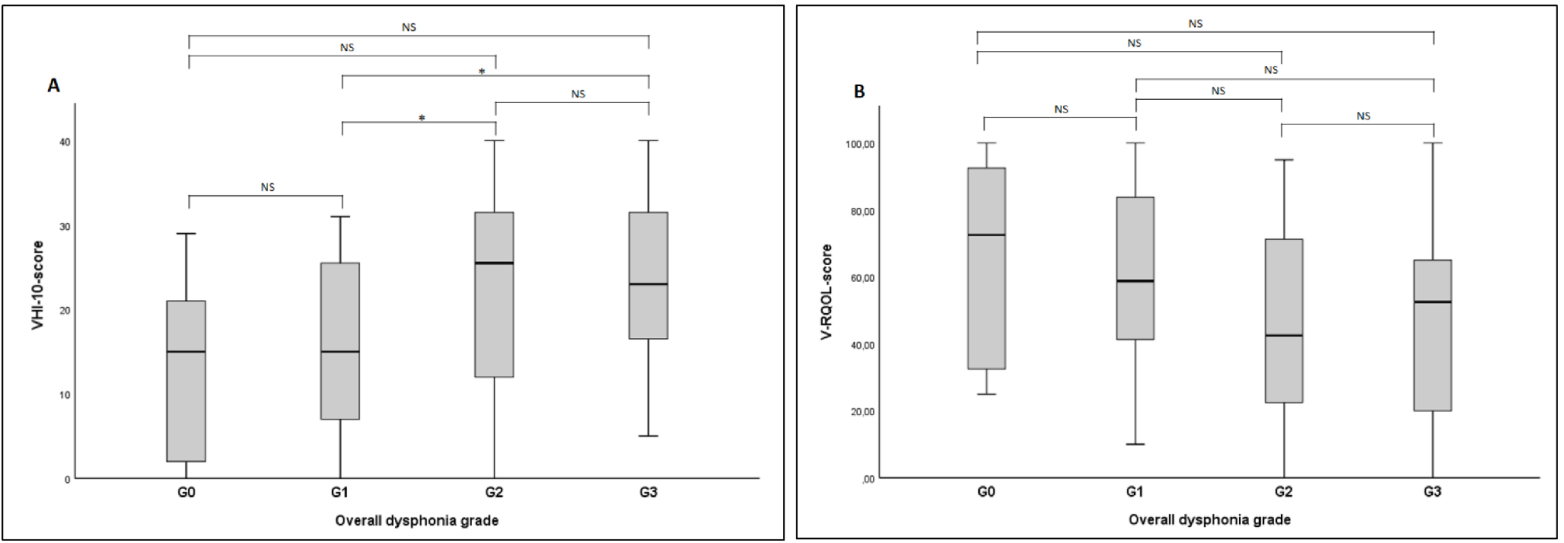
**(A)** VHI-10 and **(B)** V-RQOL scores based on dysphonia severity *Abbreviations*: VHI-10, voice handicap index-10; V-RQOL, voice-related quality of life; G, overall dysphonia grade *, *p* < 0.005 NS, not significant

The pairwise comparisons for VHI-10 significantly differed only between the groups G_1_ and G_2_ (*p* < 0.005) and the groups G_1_ and G_3_ (*p* < 0.005), while the same trend was not observed for the comparisons between the other groups. Similarly, there were not any significant differences between the V-RQOL scores of all the compared groups (Fig. 3).

**Fig. 4:**
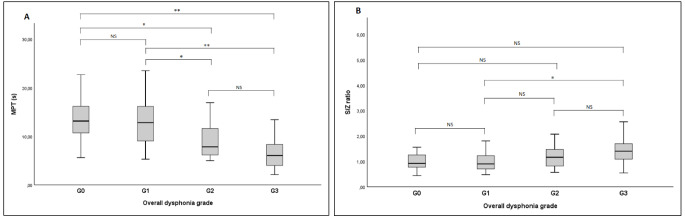
**(A)** MPT and **(B)** S/Z ratio values based on dysphonia severity Abbreviations: MPT, maximum phonation time; s, second; G, overall dysphonia grade *, *p* < 0.005 **, *p* < 0.001 NS, not significant

Significantly higher MPT values were observed in group G_0_ than in groups G_2_ (*p* < 0.005) and G_3_ (*p* < 0.001), while there were not any differences between the group G_0_ and G_1_. The MPT values in the group G_1_ were significantly higher than both the group G_2_ (*p* < 0.005) and G_3_ (*p* < 0.001). However, no significant differences were observed between the MPT values of the group G_2_ and G_3_ (Fig. 4). Comparisons for the s/z ratio revealed significant differences only between the groups G_1_ and G_3_, which was higher in the group G_3_ (*p* < 0.001), (Fig. 4).

As shown in Table 4, correlation analyses revealed significant relationships between the *G* parameter of GRBAS and all the measured variables (*p* < 0.001). Statistically significant positive correlations were found for the AVQI score (*r* = 0.642), ABI score (*r* = 0.518), VHI-10 (*r* = 0.319), and s/z ratio(*r* = 0.308), whereas negative correlations were observed for the CPPS*sv* (*r* = -0.497), CPPS*cs* (*r* = -0.646), V-RQOL (*r* = -0.270), and MPT (*r* = -0.496).

## Discussion

The voice quality of the present cohort was investigated comprehensively using a multidimensional approach, which included videolaryngostroboscopic examination, acoustic voice analyses, PROs, durational measurements, and auditory-perceptual assessment. Since auditory-perceptual evaluation of voice is considered as the “gold standard” method for discriminating the dysphonic voice from normal voice and also rating the dysphonia severity, the dysphonia grade of the subjects were determined using auditory-perceptual evaluation. After rating the severity of dysphonia with the *G* parameter of the GRBAS scale, the acoustic, PROs, and durational parameters were described for each dysphonia grade (G_0_, G_1_, G_2_, and G_3_). Further, the relationships between the dysphonia grade and all measured variables (AVQI and ABI scores, CPPS values, VHI-10 and V-RQOL scores, and MPT and s/z ratio) were investigated to reveal how these voice-related dimensions correlate with the severity of dysphonia.

The multiparametric indexes have been frequently used to discriminate normal voice from dysphonic voice [23, 54, 55]. For AVQI and ABI, the higher score means a greater severity of dysphonia, which ranges from 0 (no dysphonia) to 10 (severe dysphonia). Many studies in the literature confirmed a significant correlation between AVQI and overall voice quality, as well as ABI and perceived breathiness severity [56–58]. In line with the previous studies, we observed a significant relationship between the overall dysphonia grade and the AVQI and ABI scores. As an expected result, the AVQI and ABI scores of the severely dysphonic group (G_3_) were significantly higher than the other groups (G_0_, G_1_, and G_2_) in this study. While the AVQI scores of the moderately dysphonic group (G_2_) were significantly higher than the normal (G_0_) and slightly dysphonic (G_1_) groups, the ABI score in group G_2_ was significantly higher than the group G_0_, but not than the group G_1_. On the other hand, comparisons between the normal (G_0_) and slightly (G_1_) groups did not reveal any significant differences for both the AVQI and ABI scores.

To the best of our knowledge, there is currently no normative data on ABI for Turkish speakers to compare with the current study, but in their study, Yeşilli-Puzella et al. [59] reported the cutoff point for dysphonia in Turkish speakers as 2.345 for AVQI (v. 03.01). In our study, the mean ± SD value of AVQI in the G_0_ group was 2.96 ± 1.01, which was higher than the cutoff score of the prior study. The general patient characteristics (age, gender, diagnosis, etc.) and using different voice recording procedures (the microphone type, the mouth-to-microphone distance, and the recording environment) should be the probable reasons for these inconsistent findings between the studies. Another possible reason may be the methodology used to measure the AVQI scores. For example, in the former study, the duration of the concatenation of the voiced parts of the continuous speech had various durations between 0.27 and 7.20 s, while we used a standard 3.00 s of customized length of continuous speech procedure [46]. For these reasons, the threshold values of the AVQI and ABI indexes reported for the different dysphonia grades (G_0_, G_1_, G_2,_ and G_3_) in the present study should be interpreted with caution. Nonetheless, the clinicians at least keep in mind that the thresholds gradually increase with the severity of dysphonia. Ultimately, it was suggested that clinicians should establish their own clinical norm values ​​for both indexes to guide their diagnosis or treatment planning.

As a cepstral-based method, CPPS measurements have been reported by many studies to be a strong predictor of dysphonia for both sustained vowel and continuous speech samples [10, 60–63]. In our study, we also confirmed that the CPPS values for sustained vowel (CPPS*sv*) and connected speech (CPPS*cs*) had close relationships with dysphonia severity. Heman-Ackah et al. [10] reported in their study that the CPPS parameter was strongly related to overall voice quality in both sustained vowels (*r* = -0.80) and especially in continuous speech (*r* = -0.86). Even if we did not confirm a strong relationship between the overall voice quality and CPPS*sv* and CPPS*cs* parameters, similar to the previous study, the correlation coefficient value for connected speech (*r* = -0.646) was higher than the sustained vowel (*r* = -0.497). Therefore, as recommended in the literature, we suggested performing acoustic analyses using connected speech samples if clinically possible. Also, the present study showed significant differences between the CPPS findings of group G_0_ and groups G_2_ and G_3_. Therefore, CPPS should be used as a robust parameter, especially for discriminating perceptually normal voices from moderately or severely deviated voices.

The VHI-10 and V-RQOL scores did not reveal any significant difference in the comparisons between those with normal (G_0_) and disordered voice quality (G_1_, G_2_, and G_3_). Karnell et al. [64] reported relatively weak agreement between PROs and clinician-based judgments such as CAPE-V and GRBAS in their study. It was concluded that experiencing and considering the dysphonia severity is very different between clinicians and patients. In another study conducted by Murry et al. [64], it was indicated that the ratings of dysphonia by the clinicians were more severe than the ratings for the VRQOL by the patients. The study proposed that factors beyond those directly related to voice quality might influence patients’ responses on voice assessment scales. It is known that measuring the quality of life in patients with dysphonia depends on the patients’ voice-related perception and experience [64]. In this case, to improve the sensitivity of PROs in dysphonia severity assessments, it may be helpful to fill out the PROs together with the patients because they tend to under- or overestimate the dysphonia severity. On the other hand, alternative PROs instead of the present ones may also be more beneficial to differentiate the dysphonia severity in future studies. Overall, a comprehensive voice assessment should be conducted based on both the patient’s perspective and the clinicians’ perceptual evaluation to give valuable insight into the extent of altered vocal physiology. In line with the previous studies, we recommend incorporating both PROs and clinicians’ perceptual judgments when determining the overall dysphonia grade. Although the scores of both questionnaires and overall dysphonia grade were significantly correlated, it should be kept in mind that both correlations had small r values (VHI-10; *r* = 0.319 and V-RQOL; *r* = -0.270). Therefore, the present findings should be interpreted with caution; even similar findings were confirmed in several prior studies [64–66]. For example, Murry et al. [64] stated a moderately negative significant correlation between V-RQOL score and total GRBAS score in a cohort of patients with a chief complaint related to their voice. In another study, Fujiki and Thibeault [66] included 508 adults with voice disorders and reported a weakly positive correlation between the total VHI score and overall grade of dysphonia (*p* < 0.001, *r* = 0.32). Because the general voice-related patient-reported outcomes have their own values but also deficits in psychometric properties [27], a multidimensional approach should be considered fundamental to a complete assessment of dysphonia.

The MPT has been used in different studies for differentiation between normal and dysphonic voices [67–70]. Yu et al. [68] and Aghajanzadeh et al. [70] compared the MPT results of patients with normal, slightly deviated, moderately deviated, and severely deviated voices based on the *G* parameter of GRBAS. The results in both studies showed that MPT significantly differed from one group to another. In the present study, the MPT values significantly discriminated the normal (G_0_) and slightly deviated (G_1_) voices from both moderately (G_2_) and severely deviated (G_3_) voices. However, the same discriminant ability was not evident between the normal (G_0_) and slightly deviated voices (G_1_) and also between the moderately (G_2_) and severely (G_3_) deviated voices. The probable reason for this difference could be attributed to the characteristics of the patients included in the studies and variations in the sample sizes. For each voice clinic, it is better to use their own normative MPT data, but still, the clinicians should consider the mean ± SD MPT values obtained for each group in the present study, which decreased gradually as the dysphonia grade increased. We also found a moderately negative significant correlation (*p* < 0.001, *r* = -0.496) between MPT and overall dysphonia grade. In contrast to the present findings, Cantarella et al. [69] found no correlation between MPT and overall dysphonia severity; nevertheless, the majority of studies confirm this relationship [66, 68, 70]. Therefore, voice clinicians should be aware that MPT is associated with overall dysphonia severity, as the shorter duration of MPT can be an indicator of more severe dysphonia. In a pioneering study, Eckel and Boone [30] postulated that s/z ratio of lower than 1.4 may be an indicator of a laryngeal pathology. In our study, the s/z ratio was found above this cutoff score (mean ± SD 1.82 ± 1.81; range, 0.55–2.56) only in patients with severe dysphonia, and the results of *post hoc* comparisons showed that the discriminant power of s/z ratio was only evident in discriminating the slightly deviated (G_1_) voices from severely deviated (G_3_) voices. In addition, a weakly positive significant correlation was observed between the s/z ratio and overall dysphonia grade. It can be concluded from the present study that the s/z ratio alone is not sufficient to differentiate the dysphonia severity, and therefore, careful attention should be paid by the clinicians while interpreting the s/z ratio for clinical decision-making. In this direction, as reported by Joshi [71], we also recommend using the s/z ratio in combination with other instrumental measures to enhance the accuracy of laryngeal aerodynamics.

The present study has several weaknesses. First, the design of the present study limits the ability to assess the progression of dysphonia and the impact of interventions due to the absence of longitudinal data. Second, professional voice users, a key population affected by dysphonia, were excluded from the study. It is known that both the VHI-10 and V-RQOL questionnaires have been performed on patients from a wide range of backgrounds. However, it is also known that professional voice users experience differential impacts on voice impairment compared with non-professional voice users, which may not be fully reflected in the VHI-10 and V-RQOL. Instead, using a more specific questionnaire (i.e., Singing Voice Handicap Index) would give more robust findings for the target population. Third, since the durational measurements (MPT and s/z ratio) may be used as indirect measures of certain aerodynamic parameters, more objective respiratory/aerodynamic measurements (i.e., vital capacity, subglottal pressure, laryngeal airway resistance, mean expiratory airflow, aerodynamic efficiency, etc.) are recommended for future studies. Fourth, reliability analyses for the G parameter were conducted with two raters. More robust findings could be achieved by performing auditory-perceptual assessments using a larger number of raters. Lastly, because the present findings are restricted to Turkish-speaking patients, future longitudinal studies with diverse dysphonia populations would be beneficial to ensure broader generalizability of the results and to address the other limitations of the present study design.

## Conclusions

Based on the present findings, it was suggested that measuring the AVQI and ABI scores, the CPP values of sustained vowel (CPP*sv*) and connected speech (CPP*cs*) samples, the VHI-10 scores, and the MPT values should provide valuable information for overall dysphonia severity. The mentioned parameters may be considered to discriminate, especially the moderate or severe dysphonia from the normal and/or slightly deviated voices. Future studies, including a larger cohort of patients with various etiology of dysphonia are needed to confirm the present findings.

## Data Availability

All data generated and/or analyzed during the current study are available from the corresponding author upon reasonable request.
